# Re-emergence of Gamma-like-II and emergence of Gamma-S:E661D SARS-CoV-2 lineages in the south of Brazil after the 2021 outbreak

**DOI:** 10.1186/s12985-021-01690-1

**Published:** 2021-11-17

**Authors:** Mauro M. Oliveira, Michelle O. Schemberger, Andreia A. Suzukawa, Irina N. Riediger, Maria do Carmo Debur, Guilherme Becker, Paola Cristina Resende, Tiago Gräf, Eduardo Balsanelli, Valter Antônio de Baura, Emanuel M. de Souza, Fábio O. Pedrosa, Lysangela R. Alves, Lucas Blanes, Sheila Cristina Nardelli, Alessandra M. Aguiar, Letusa Albrecht, Dalila Zanette, Andréa R. Ávila, Luis Gustavo Morello, Fabricio K. Marchini, Hellen G. dos Santos, Fabio Passetti, Bruno Dallagiovanna, Helisson Faoro

**Affiliations:** 1 Laboratório de Ciências e Tecnologias Aplicadas Em Saúde, Instituto Carlos Chagas, FIOCRUZ, Curitiba, Paraná Brazil; 2 Laboratório de Biologia Básica de Células Tronco, Instituto Carlos Chagas, FIOCRUZ, Curitiba, Paraná Brazil; 3 Laboratório Central do Estado do Paraná, LACEN, Curitiba, Paraná Brazil; 4 Laboratórios de Vírus Respiratórios e do Sarampo (LVRS), Instituto Oswaldo Cruz, FIOCRUZ, Rio de Janeiro, Rio de Janeiro Brazil; 5 Instituto Gonçalo Moniz, FIOCRUZ, Salvador, Bahia Brazil; 6 Departamento de Bioquímica e Biologia Molecular, Universidade Federal Do Paraná, Curitiba, Paraná Brazil; 7 Laboratório de Regulação da Expressão Gênica, Instituto Carlos Chagas, FIOCRUZ, Curitiba, Paraná Brazil; 8 Laboratório de Pesquisa em Apicomplexa, Carlos Chagas Institute, FIOCRUZ, Curitiba, Paraná Brazil; 9 Instituto Carlos Chagas, FIOCRUZ, Curitiba, Paraná Brazil

**Keywords:** Phylogenetic analyses of SARS-CoV-2, VOC Gamma, Gamma-like-II and new mutations

## Abstract

**Background:**

We report a genomic surveillance of SARS-CoV-2 lineages circulating in Paraná, southern Brazil, from March 2020 to April 2021. Our analysis, based on 333 genomes, revealed that the first variants detected in the state of Paraná in March 2020 were the B.1.1.33 and B.1.1.28 variants. The variants B.1.1.28 and B.1.1.33 were predominant throughout 2020 until the introduction of the variant P.2 in August 2020 and a variant of concern (VOC), Gamma (P.1), in January 2021. The VOC Gamma, a ramification of the B.1.1.28 lineage first detected in Manaus (northern Brazil), has grown rapidly since December 2020 and was thought to be responsible for the deadly second wave of COVID-19 throughout Brazil.

**Methods:**

The 333 genomic sequences of SARS-CoV-2 from March 2020 to April 2021 were generated as part of the genomic surveillance carried out by Fiocruz in Brazil Genomahcov Fiocruz. SARS-CoV-2 sequencing was performed using representative samples from all geographic areas of Paraná. Phylogenetic analyses were performed using the 333 genomes also included other SARS-CoV-2 genomes from the state of Paraná and other states in Brazil that were deposited in the GISAID. In addition, the time-scaled phylogenetic tree was constructed with up to 3 random sequences of the Gamma variant from each state in Brazil in each month of 2021. In this analysis we also added the sequences identified as the B.1.1.28 lineage of the Amazonas state and and the Gamma-like-II (P.1-like-II) lineage identified in different regions of Brazil.

**Results:**

Phylogenetic analyses of the SARS-CoV-2 genomes that were previously classified as the VOC Gamma lineage by WHO/PANGO showed that some genomes from February to April 2021 branched in a monophyletic clade and that these samples grouped together with genomes recently described with the lineage Gamma-like-II. Additionally, a new mutation (E661D) in the spike (S) protein has been identified in nearly 10% of the genomes classified as the VOC Gamma from Paraná in March and April 2021.Finally, we analyzed the correlation between the lineage and the Gamma variant frequency, age group (patients younger or older than 60 years old) and the clinical data of 86 cases from the state of Paraná.

**Conclusions:**

Our results provided a reliable picture of the evolution of the SARS-CoV-2 pandemic in the state of Paraná characterized by the dominance of the Gamma strain, as well as a high frequencies of the Gamma-like-II lineage and the S:E661D mutation. Epidemiological and genomic surveillance efforts should be continued to unveil the biological relevance of the novel mutations detected in the VOC Gamma in Paraná.

**Supplementary Information:**

The online version contains supplementary material available at 10.1186/s12985-021-01690-1.

## Introduction

Coronavirus disease 2019 (COVID-19) is caused by severe acute respiratory syndrome coronavirus 2 (SARS CoV-2). Currently, Brazil ranks third in absolute numbers of cases, second in absolute numbers of deaths and ninth in number of deaths per million inhabitants (https://covid19.who.int/). To date, Paraná has almost 1 million laboratory confirmed COVID-19 cases and more than 195.9 deaths per 100,000 habitants [1]. On the other hand, immunization efforts have progressed slowly: only 10% of Paraná citizens have been fully vaccinated thus far [2]. The state of Paraná is strategically located in southern Brazil due to its borders with other Brazilian states (São Paulo, Santa Catarina and Mato Grosso do Sul) and with Argentina and Paraguay. This scenario of uncontrolled coronavirus spread may favor the emergence of potentially more contagious variants. Genomic surveillance efforts have been allowed to monitor the emergence and spread of new SARS CoV-2 variants worldwide. Two lineages, the B.1.1.28 and B.1.1.33 variants, were dominant during the first wave of COVID-19 in Brazil [3, 4]. However, the number of people infected with the VOC Gamma (P.1), a ramification of the B.1.1.28 lineage first detected in Manaus (northern Brazil) that harbors the E484K and N501Y mutations in the spike (S) protein [5], has grown rapidly since December 2020 and was thought to be responsible for the deadly second wave of COVID 19 throughout Brazil [6–8]. Compared to the first wave, more cases and deaths were registered in a shorter period of time during the second wave. According to official Brazilian Ministry of Health data, from the beginning of the pandemic until the end of November 2020, 282,645 cases of infection were registered in Paraná, which resulted in 6,160 deaths [9]. Between December 2020 and April 2021, an additional 663,528 cases and 16,160 deaths were recorded [10]. Although many factors may have contributed to this scenario, the introduction and spreading of new SARS-CoV-2 variants in the state of Paraná and the relationship between case severity and the prevalence of the VOC Gamma have not yet been investigated.

In this study, we carried out an analysis of 333 SARS-CoV-2 genomes of strains circulating in Paraná from February 2020 to April 2021. Our results demonstrate a cocirculation of the dominant VOC Gamma and the recently described Gamma-like-II (P.1-like-II) lineage [11]. Notably, a new S:E661D mutation present in approximately 10% of the VOC Gamma genomes from March and April 2021 was identified. Mutations in the S protein are of particular interest because they may favor viral immune evasion and/or alter the efficacy of viral-cell interactions. Finally, we address the relationship between the prevalence of the Gamma variant, the severity of the cases and the age group strata.

## Materials and methods

### Selection and recovery of samples

**Fig. 1:**
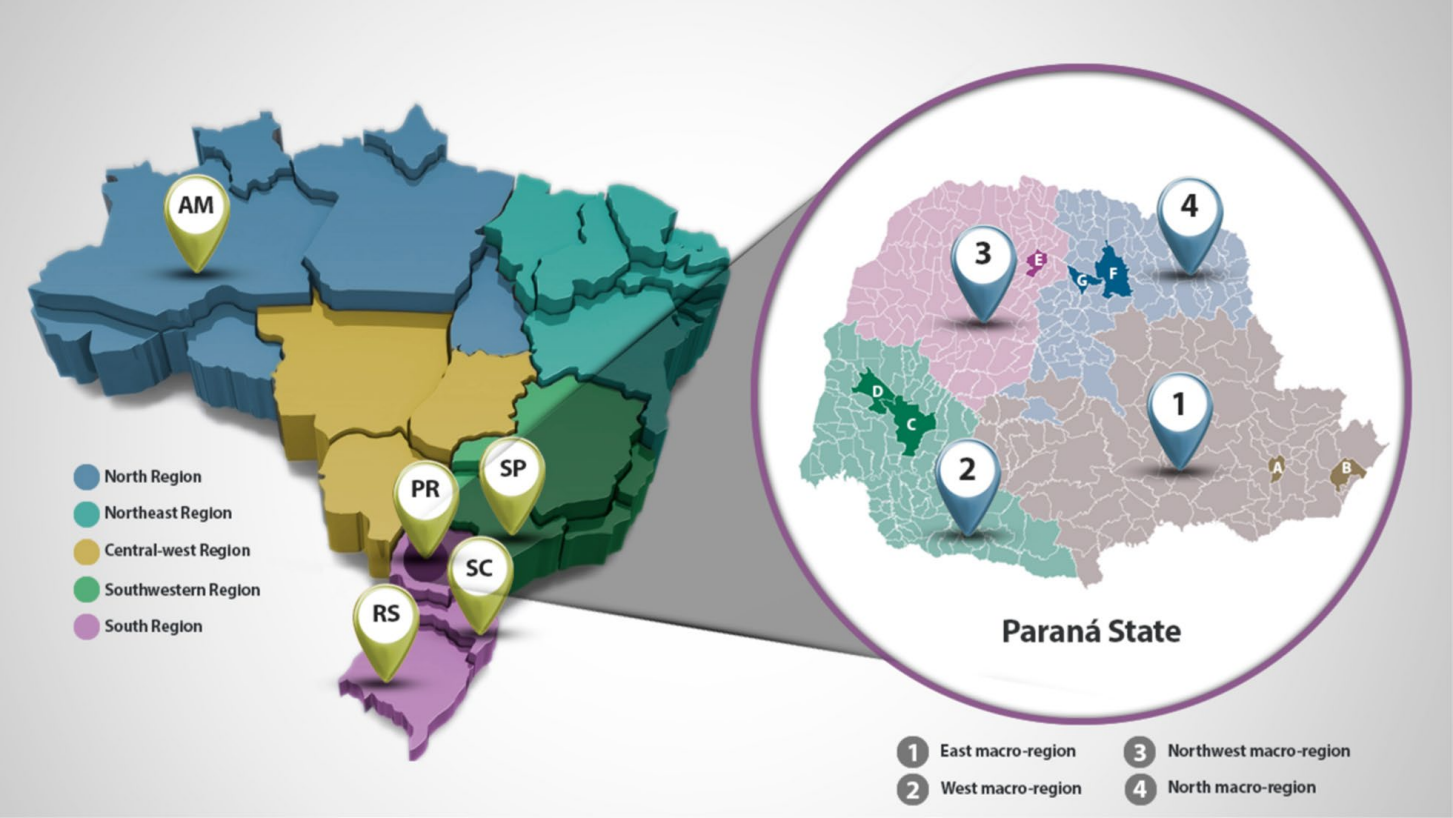
Sampling of SARS-CoV-2-positive patients was performed according to Paraná’s Secretary of Health official macroregional division. The map of Brazil is shown on the left. The color code represents the 5 regions of the country. The states of Amazonas (AM), São Paulo (SP), Paraná (PR), Santa Catarina (SC) and Rio Grande do Sul (RS) are indicated. The map of Paraná is shown on the right, and each macroregion was represented by at least one host city, as follows: (1) the east macroregion was represented by **a** Curitiba and **b** Paranaguá; (2) the west macroregion was represented by **c** Cascavel and **d** Toledo; (3) the northwest macroregion was represented by **e** Maringá; and (4) the north macroregion was represented by **f** Londrina and **g** Apucarana cities

The 333 genomic sequences of SARS-CoV-2 from March 2020 to April 2021 were generated as part of the genomic surveillance carried out by Fiocruz in Brazil GenomahcovFiocruz. The genomic sequences specifically identified in April 2021 were used to investigate the relationship between the frequency of the Gamma variant, the age group (patients younger or older than 60 years old) and the clinical severity (mild or severe cases), in addition to surveillance. For this purpose, SARS-CoV-2 samples were obtained from the two main laboratories responsible for the diagnosis of COVID-19 in the state of Paraná: the LACEN-PR (the Central Laboratory of the State of Paraná) and the LAC (COVID-19 Diagnostic Support, the Paraná State Unit—IBMP/Fiocruz). From the LACEN laboratory, samples of SARS-CoV-2 were taken from severe cases, while samples representing mild cases were obtained from the LAC. We aimed to obtain representative samples from all geographic areas of Paraná, and we considered the official division of the state into macroregions, each one with host cities: (1) EAST macroregion—host cities Curitiba and Paranaguá; (2) WEST macroregion—host cities Cascavel and Toledo; (3) NORTHWEST macroregion—host city Maringá; and (4) NORTH macroregion—host cities Londrina and Apucarana (Fig. 1).

An equal allocation of samples were obtained in each clinical severity, host city/macroregion and age group strata. Thus, mild and severe cases were represented by 48 samples each, and a same number of samples were taken from each host city/microregion. Also, for each geographic region, an equal allocation of samples was used for each age strata (5 samples each). In the eastern macroregion, the municipality of Curitiba was considered separately from that of Paranaguá: the capital (Curitiba) contributed 20 samples (10 per laboratory and 5 per age group), and Paranaguá contributed 16 samples (8 per laboratory and 4 per age group). To assess whether there is an association between the frequency of the Gamma variant and the age group or the severity of COVID-19, we used a Fisher’s exact test at the 5% significance level. Additionally, we estimated the odds ratio (OR) and the corresponding 95% confidence interval (95% CI) considering age less than 60 years and mild case severity as the reference categories (OR=1.00).

### SARS-CoV-2 RNA purification and sequencing

Nasopharyngeal and pharyngeal swabs from infected patients (Ct < 25) were suspended in viral transport medium and maintained at -80 °C. Viral RNA was purified from these samples using the QIAamp ViralRNA Purification Kit (Qiagen). The purified RNA was converted to cDNA, and the viral genome was amplified by PCR using the Artic protocol [12]. The genome was sequenced on the Illumina MiSeq platform in the 2x300 bp paired-end configuration. Variants were identified after read mapping in the reference genome for SARS-CoV-2 (RefSeq accession number NC 045512), and the analysis was performed on the Pangolin and Nextclade platforms. This study was approved by the Hospital do Trabalhador/SES/PR Ethics Committee: CAAE 31650020.5.0000.5225.

### SARS-CoV-2 genome assembly and variant calling

The bioinformatics analyzes were carried out at the Fiocruz Bioinformatic Platform Highz server (Fiocruz Rio—RPT04). The reads were trimmed with Trimmo matic v0.39 [13] and assembled using the tools SPAdes v3.14.1 [14] and MEGAHIT v1.2.9 [15]. The scaffolds were built with RagTag v1.1.1 [16], ABACAS v1.3.1 [17] using the SARS-CoV-2 isolate Wuhan-Hu-1 as a template (NC 045512 RefSeq). Using this approach, we generated consensus sequences with a mean of 95% coverage (QUAST v5.0.2) [18]. To assign the viral lineages, we used the nomenclature proposed by Ram baut et al [19], using the Pangolin standalone (pangolin v2.4.2 and pangoLEARN 2021-04-28). In parallel, we also assigned the viral lineages using the Nextclade web application (https://clades.nextstrain.org).

### Phylogenetic analysis

Phylogenetic analyses were performed using the 333 genomes generated as part of the Fiocruz COVID-19 Genomic Surveillance Network but also included other SARS-CoV-2 genomes from the state of Paraná and other states in Brazil that were deposited in the GI SAID (Additional file 8: Appendix S1). We retrieved all high-quality (< 5% of N) complete (> 29 kb) SARS-CoV-2 genomes of lineages B.1.1.28, B.1.1.33, Gamma and P.2 sampled in the state of Paraná, the B.1.1.28 lineage sam pled in the state of Amazonas, and the Gamma vari ant sampled in the states of Amazonas, Santa Catarina, Rio Grande do Sul and from other regions of Brazil that were available on the GISAID [20] as of May 14, 2021. The set of sequences was divided into two datasets: (1) Paraná sequences (n = 396) and (2) Paraná + Out Paraná sequences (n = 354). The two groups of sequences were aligned using MAFFT v7.475 [21]. The datasets were subjected to a maximum likelihood (ML) and a phylogenetic analysis using IQ TREE v2.1.2 [22] under the GTR + G4 + F nucleotide substitution model, and branch support was assessed by the approximate likelihood-ratio test based on the Shimodaira–Hasegawa-like procedure (SH-aLRT) with 1,000 replicates. Trees were visualized on the web application Interactive Tree Of Life (iTOL v5) [23]. To highlight the mutations and categorize them by the frequency, genomic location and effect on protein sequences, we performed single nucleotide polymorphisms (SNPs) analysis with Snippy tools and Coronapp. The SNPs were assessed in each sample using the Wuhan-Hu-1 genome sequence as the reference. The results of the two tools were considered positive when the SNPs observed were predicted in the two tools.

### Time-scaled phylogenetic analysis

The time-scale phylogenetic tree was built using the Bayesian Markov Chain Monte Carlo (MCMC) approach implemented in BEAST 1.10.4 with the BEAGLE v3.1.0 library to optimize the computational time. The Bayesian tree was reconstructed using the HKY substitution model and the gamma distribution to account for rate heterogeneity across sites. The tree prior with parameter was set up using the co alescent Bayesian skyline model to create a molec ular clock using the lognormal relaxed-clock model with an exponential substitution rate (8 × 10^−4^ sub stitutions/site/year). Two MCMC chains were run for 200 million generations and then were combined to ensure stationarity and good mixing. Convergence (effective sample size > 200) in parameter estimates was assessed using TRACER v1.7.2. The maximum clade credibility (MCC) tree was summarized with TreeAnnotator v1.10.4 ML, and MCC trees were vi sualized using FigTree v1.4.4 [24, 25]. In parallel, we built phylogenetic trees on a time scale with the same SARS-CoV-2 samples using the TreeTime tool with default parameters [26]. The time-scaled phyloge netic tree was performed using 2 sets of the SARS-CoV-2 sequence. In the group 1, the dataset was constructed with up to 3 random sequences from each state in Brazil in each of the months of 2021 (Period 2021-01-01 to 2021-05-14; GISAID). Furthermore, the sequences were identified as the B.1.1.28 lineage from the Amazonas state (n = 15) and the Gamma-like-II lineage genome from different Brazilian states: Paraná (n = 20), Santa Catarina (n = 30), São Paulo (n = 14), Rio de Janeiro (n = 5), Rio Grande do Sul (n = 5), Minas Gerais (n = 3), Espírito Santo (n = 1) and Alagoas (n = 1). In group 2, the dataset was constructed with up to 5 random sequences from the state of Paraná in each month of 2021 (Period 2020- 03-01 to 2021-09-23; GISAID). In addition, the sequences were identified as the Gamma lineage from the Amazonas state (n = 1), the B.1.1.28 lineage genome Paraná (n = 16), Gamma lineage genome Paraná (n = 18) and Gamma-like-II lineage genome Paraná (n = 48—P.1.2 = 18, P.1.3 = 4, P.1.4 = 2, P.1.7 = 20, P.1.9 = 4).

## Results

**Fig. 2:**
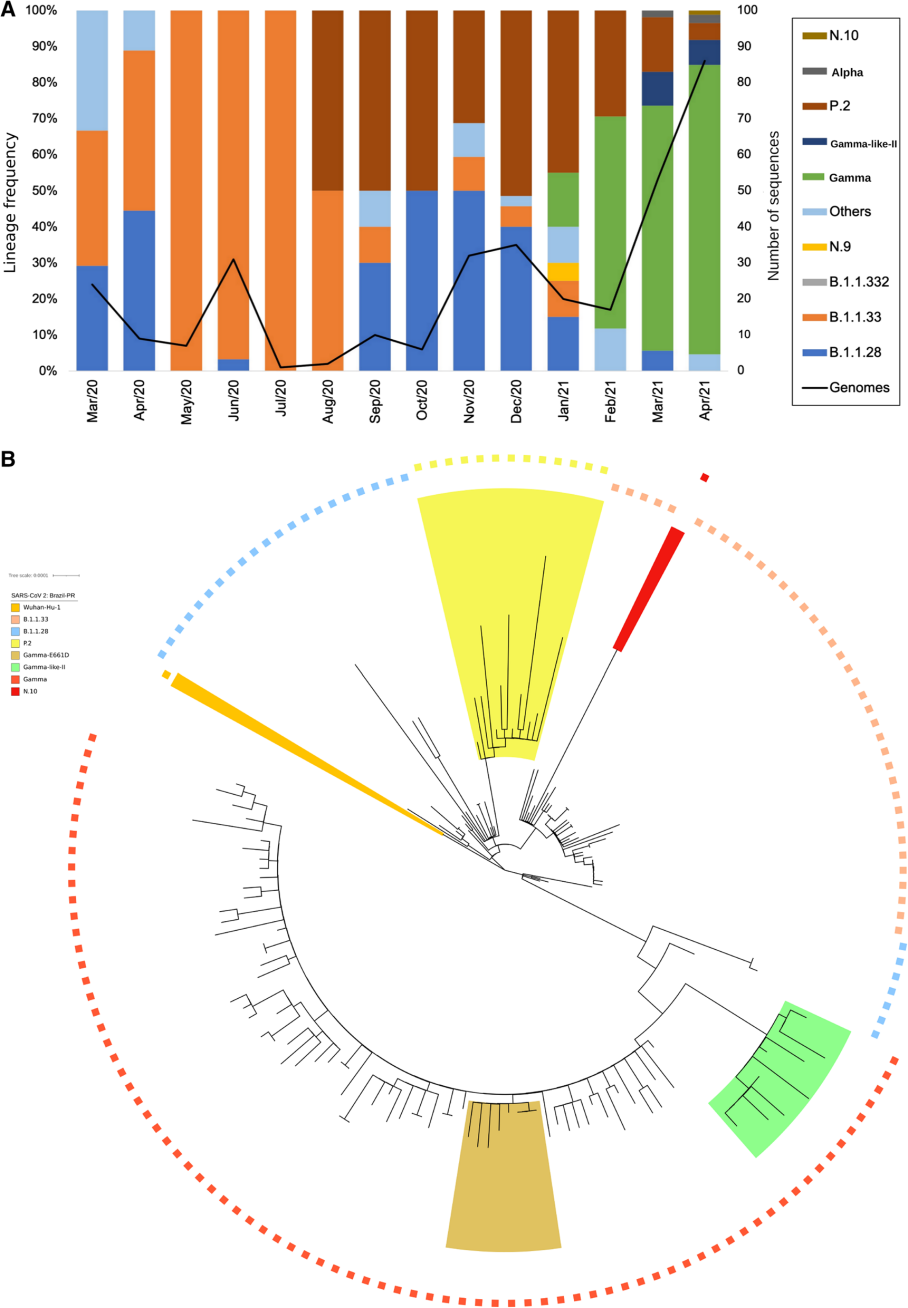
SARS-CoV-2 variants in Paraná state identified by genome sequencing. **a** Frequency and temporal distribution of the SARS-CoV-2 variant from February 2020 to April 2021. Information on the number of sequenced genomes and identification of variants from February 2020 to February 2021 were retrieved from the website genomahcov.fiocruz.br. The”Genomes” line indicates the number of representative sequences that were used to estimate the variant frequency at each time point. **b** Phylogenetic analysis of SARS-CoV-2 genomes identified in the state of Paraná. High-quality genomic sequences (n < 5%) were aligned using Mafft software in the default configuration. The phylogenetic tree was built using the maximum likelihood method in IQTree software and visualized in IToL. The scale of the phylogenetic branches is given as substitutions per nucleotide site. Each symbol represents a SARS-CoV-2 lineage, as shown in the legend. The lineage N.10 is highlighted in red; the lineage P.2 is highlighted in light yellow; the Gamma-like-II genomes are highlighted in light green; Gamma sequences harboring S:E661D are highlighted in shadow golden and Wuhan-Hu-1 is highlighted in orange

The analysis of the accumulated frequencies from March 2020 to April 2021 (Fig. 2) showed the B.1.1.33 and B.1.1.28 variants as the main variants that predominated in the state in 2020. Lineage P.2, derived from the B.1.1.28 variant, was identified in the state in August 2020 and had surpassed the previous two, reaching a frequency of 50% of the total genomes sequenced in September 2020 (Fig. 2). This scenario started to change in December 2020 with the emergence of the VOC Gamma in the Brazilian state of Amazonas and the subsequent dissemination in the state of Paraná. The Gamma variant was identified in Paraná in January 2021 (15% of the genomes), and in February 2021, it already corresponded to 58% of the sequenced genomes, replacing the P.2 variant as the dominant variant (Fig. 2). In April, the Gamma variant was identified in 87.2% of all the sequenced genomes.

To refine our knowledge about the prevalence of the VOC Gamma in the state of Paraná and its relationship with age strata (patients < 60 or ≥ 60 years old) and clinical severity (mild or severe COVID-19 cases), we selected a cohort of 96 patients who tested positive for COVID-19 (Ct < 25) in April 2021 to sequence the genome of the virus that infected them. Of the 86 genomes successfully sequenced, the variant Gamma was the most frequent (n = 75; 87.2%), followed by the P.2 variant (n = 4; 4.7%) (Additional file 1: Figure S1). The VOC Gamma was predominant in all macroregions, ranging from 58.8% in the western region to 100% in Curitiba (eastern region), and in both age groups (Additional file 1: Figure S1), although a higher diversity of variants was observed in the < 60-year-old stratum (Additional file 3: Figure S3). Regarding the sampling strata established, we did not observe any association between the frequency of the Gamma variant and age group (*p* value 0.33) or the case severity (*p* value 0.75) (Additional files 3, 4: Figures S3 and S4) in this cohort.

**Fig. 3:**
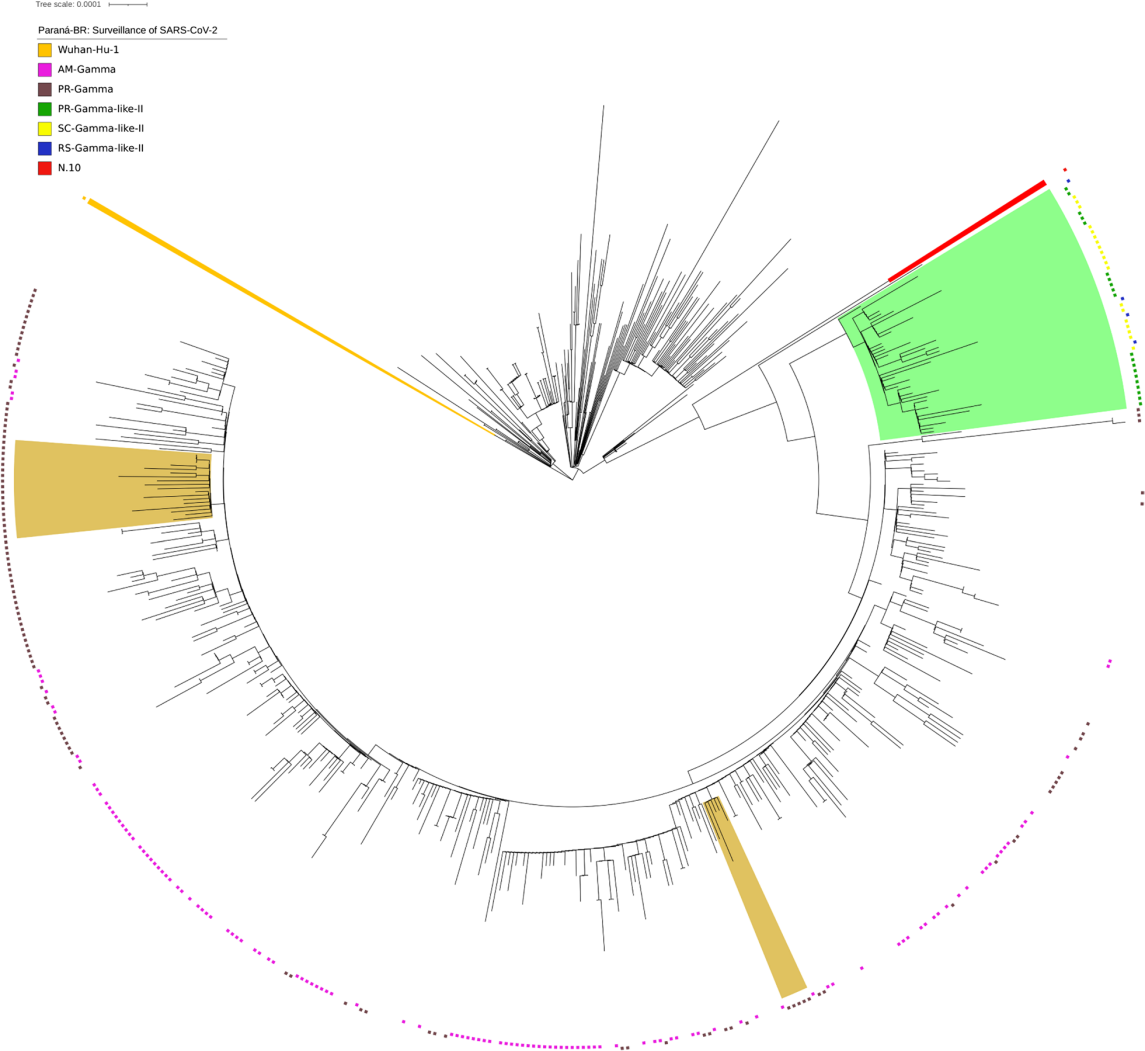
Phylogenetic tree of the VOC Gamma strain isolated in the State of Paraná from 2020-03-05 to 2021-04-28. High-quality genomic sequences (n < 5%) were aligned using Mafft software in the default configuration. The phylogenetic tree was built using the maximum likelihood method in IQTree software and visualized in IToL. Phylogenetic tree highlighting the Gamma variant containing the sequences of Amazonas, Santa Catarina and Rio Grande do Sul and closely related sequences, including those of the subclad Gamma-like-II. The aLRT support values are indicated in key branches, and the scale of the phylogenetic branches is given as substitutions per nucleotide site. Each symbol represents a SARS-CoV-2 lineage, as shown in the legend. The lineage N.10 is highlighted in red; the Gamma-like-II genomes are highlighted in light green; Gamma sequences harboring S:E661D are highlighted in shadow golden and Wuhan-Hu-1 is highlighted in orange

A phylogenetic analysis using the 333 SARS-CoV 2 genomes of the samples from the state of Paraná confirmed the previous analyses [3, 4], with variants B.1.1.28 and B.1.1.33 being the most dominant in 2020 (Fig. 2). We also identified a group of 11 genomes, 5 from March and 6 from April 2021, which were previously classified as the Gamma lineage by WHO/PANGO but truly constituted a separate clade related to the Gamma variant (Fig. 2). The divergent group had lineage defining mutations compatible with the recently described lineage of the Gamma like-II lineage [11]. These samples were mainly collected in the host cities Toledo and Cascavel from the West macroregion during the months of March and April 2020. Notably, in this macroregion, we found a higher Gamma-like-II lineage prevalence (29.4%) and a lower Gamma variant (58.8%) prevalence in the cohort analysis conducted in April 2021 (Additional file 2: Figure S2). No Gamma-like-II lineage genomes were retrieved from the northern or northwestern regions of Paraná. To deepen the understanding of the presence of the Gamma-like-II lineage in the state of Paraná, we recovered the genomic sequences identified as the Gamma-like-II lineage in the other two states in the southern region of Brazil [11], Rio Grande do Sul (n = 4) and Santa Catarina (n = 30), as well as genomic sequences of the true VOC Gamma identified in these two states. Santa Catarina was of particular interest because of its geographical proximity to Paraná and because it was the Brazilian state where most of the Gamma-like-II genomes were recovered in previous studies [11]. We also included the Gamma variant genome sequences from the state of Amazonas, where it first emerged, and other Gamma variant genomes from Paraná available in the GISAID. The phylogenetic analysis involving this set of genomes supported the identification of representatives of the Gamma-like-II lineage in the state of Paraná (Fig. 3), since they grouped together with genomes previously identified as the Gamma-like-II lineage from the states of Santa Catarina and Rio Grande do Sul but were separate from the branch composed of the VOC Gamma genomes (SH-aLRT =99.5%). Additionally, the number of the Gamma-like-II lineage genomes from Paraná increased from 11 to 20 (Fig. 3), with the additional genomes corresponding to samples from March and April. An independent cluster was comprised solely by the Gamma-like-II lineage genomes from Paraná, which indicated a possible local transmission of this lineage.

**Fig. 4:**
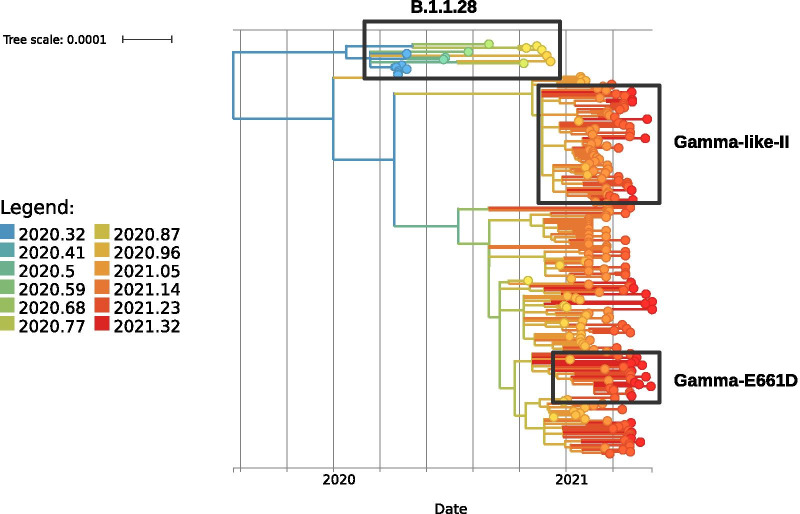
Time-scaled phylogenetic trees of SARS-CoV-2. The picture shows a time-stamped phylogeny of the Gamma lineage in the State of Paraná in comparison with sets of Gamma sequences from the States of Amazonas, Santa Catarina and Rio Grande do Sul. In the figure, the length of the branches corresponds to years and the number of mutations, and these are colored according to the legend of colors. In addition, the SARS CoV-2 reference sequence, the Gamma-like-II sequence and the Gamma sequence group with the spike protein mutation S:E661D are highlighted

The current hypothesis is that the Gamma and Gamma-like-II lineages diverged from a common ancestor [11] at a time when the mutations were arising and accumulating. To understand this question, we selected a representative group of genomes to perform a time-scaled phylogenetic tree analysis. The time-scaled phylogenetic tree (Fig. 4 and Additional file 5: Figure S5) supports the hypothesis that the Gamma-like-II and Gamma lineages have closer phylogenetic relationships than the other variants but belong to distinct clades and accumulate more mutations than the B.1.1.28 lineage from Amazonas. Our results also reinforce that the Gamma-like-II lineage and the VOC Gamma probably diverged in late August/early September (Fig. 4 and Additional file 5: Figure S5). Previous analysis based on SARS-CoV-2 genomes from Brazil obtained up until March 2021 indicated that the Gamma-like-II lineage was mainly distributed in the south and south east regions of Brazil, with a high prevalence in the state of Santa Catarina but with a unique representative from the state of Paraná [11]. Here, including the finding of novel genomic sequences, we verified that the Gamma-like-II lineage is also present in the western region of the state of Paraná at a relatively high frequency. Of note, the first representative of the Gamma-like-II genome in Paraná was identified in February 2021, while the more recent genome was found in April 2021. From this analysis, we demonstrate that the genomes classified as the Gamma-like-II lineage in the states of Paraná, Rio Grande do Sul and Santa Catarina emerged after the emergence of the Gamma variant. It would be expected that genomes referring to the Gamma-like-II lineage were found among the sequenced genomes in the state of Amazonas, which did not occur.

**Fig. 5:**
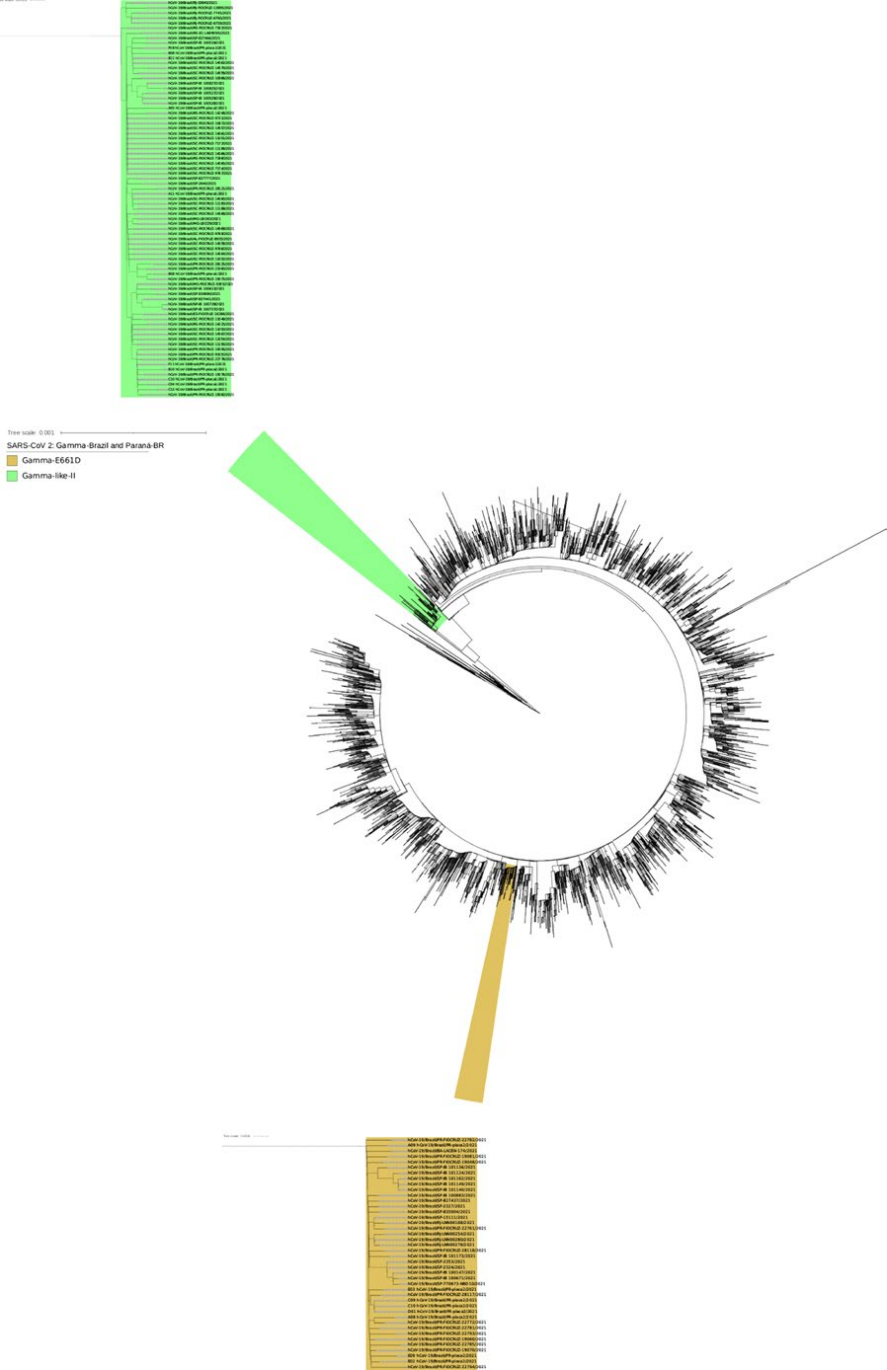
Phylogenetic tree of the Brazilian VOC Gamma. VOC Gamma genomic sequences from all Brazilian states, produced by the Fiocruz genomic network or retrieved from GISAID, were aligned using MAFFT and used to construct a phylogenetic tree. Highlighted in the boxes are the genomes of the Gamma-like-II lineage (green) and the Gamma -E661D mutation (golden). The states from which the sequences originated were represented as PR-Paraná; SC-Santa Catarina; RS-Rio Grande do Sul; SP-São Paulo; RJ-Rio de Janeiro; MG-Minas Gerais; ES-Esp´ırito Santo; AL-Alagoas; BA-Bahia

The analyses of the phylogenetic tree constructed with the Gamma sequences from Paraná state (Fig. 2) revealed the existence of a small cluster formed by only 8 Gamma genomes. The analysis of mutations in these 8 genomes revealed an additional substitution in the 661 amino acid residue of the S protein in this group of Gamma variants (Table 1). A glutamic acid residue is changed to an aspartic acid residue (S:E661D). When we exclusively considered the cohort of patients analyzed in April 2021, this mutation occurred in 8 of the 75 (10.8%) Gamma genomes from Paraná, with particular prevalence in the east ern and northern macroregions: approximately 25% of the VOC Gamma samples from Curitiba (5/20) and 3/12 samples from Londrina and Apucarana harbored the S:E661D mutation. In March 2021, the number of Gamma genomes with this mutation was 3 of the 41 analyzed genomes (7.3%) (data not shown). This result suggests that the S:E661D mutation increases in frequency over time. Among the 8 cases identified in April 2021 carrying the Gamma variant with the E661D mutation, one death was reported, 6 patients were hospitalized for more than a month, and only one patient was cured. To determine the frequencies of the Gamma-like-II lineage and the S:E661D mutation in other Brazilian states, we constructed a phylogenetic tree with all Gamma genomes sequenced in Brazil (n=3,648) (Fig. 5). As previously described [11], the Gamma-like-II lineage is distributed mainly in the south and south east regions of Brazil, with a higher prevalence in the states of Paraná and Santa Catarina. Related to the S:E661D mutation, we found, in addition to those genomes from Paraná, 25 genomes whose S protein contained the mutation, S:E661D, all from 2021. These were from Minas Gerais (n=1), São Paulo (n=19), Bahia (n=1) and Rio de Janeiro (n=4) (Fig. 5). When we considered the frequency in relation to the total number of Gamma variants identified, the E661D mutation appeared in 10.24% of the genomes from Paraná, followed by Minas Gerais (2.94%), Bahia (1.92%), São Paulo (0.90%) and Rio de Janeiro (0.86%). We also highlight the identification of 2 high-quality genomes of the variant Alpha (B.1.1.7), first identified in the United Kingdom, and one high quality genome of the N.10 variant, recently identified in the Brazilian state of Maranhão [27]. The N.10-MA variant (a term used in this article to differentiate it from the variant found in Paraná, N.10-PR) was described as a variant of interest (VOI), was derived from the B.1.1.33 variant, and carried important mutations in the S protein, including the S:V445A and S:E484K mutations. A more detailed analysis of the mutations that occurred in the N.10-PR variant revealed one ad ditional mutation in the S protein, S:W152C, which is absent in the N.10-MA variant. The S:W152C mutation occurs in the N-terminal domain (NTD) of the S protein and was previously described in another VOI, named B.1.429 (CAL.20C) (Additional file 6: Table S1). Of note, there was no evidence for the circulation of the B.1.617 variant (first observed in India) or the B.1.351 variant (first observed in South Africa) in Paraná. However, in June 2021, the State Secretary of Health of Paraná reported the identification of 3 cases of COVID-19 from Apucarana, which were caused by the variant B.1.617 [28]. These infections occurred in April (the same period of sample collection of this study), which suggests that the VOC B.1.617 was already present but had not broadly disseminated in the state by that time.

**Table 1:** Comparison between mutations identified in variant Gamma described in Amazonas, Santa Catarina and Rio Grande do Sul (Gamma-AM, Gamma-SC and Gamma-RS) and in eight Gamma samples from Paraná (Gamma-PR)

Protein	Gamma-PR								Gamma-SC	Gamma-AM	Gamma-RS
	C09	D01	A09	A08	E09	C10	E02	E03			
N	P80R	P80R	P80R	P80R	P80R	P80R	P80R	P80R	P80R	P80R	P80R
	R203K	R203K	R203K	R203K	R203K	R203K	R203K	R203K	R203K	R203K	R203K
	G204R	G204R	G204R	G204R	G204R	G204R	G204R	G204R	G204R	G204R	G204R
ORF1a	S1188L	S1188L	S1188L	S1188L	S1188L	S1188L	S1188L	S1188L	S1188L	S1188L	S1188L
	K1795Q	K1795Q	K1795Q	K1795Q	K1795Q	K1795Q	K1795Q	K1795Q	K1795Q	K1795Q	K1795Q
ORF1b	P314L	P314L	P314L	P314L	P314L	P314L	P314L	P314L	P314L	P314L	P314L
	E1264D	E1264D	E1264D	E1264D	E1264D	E1264D	E1264D	E1264D	E1264D	E1264D	E1264D
ORF3a	S253P	S253P	S253P	S253P	S253P	S253P	S253P	S253P	S253P	S253P	S253P
ORF8	E92K	E92K	E92K	E92K	E92K	E92K	E92K	E92K	E92K	E92K	E92K
ORF9b	Q77E	Q77E	Q77E	Q77E	Q77E	Q77E	Q77E	Q77E	Q77E	Q77E	Q77E
Spike	L18F	L18F	L18F	L18F	L18F	L18F	L18F	L18F	L18F	L18F	L18F
	T20N	T20N	T20N	T20N	T20N	T20N	T20N	T20N	T20N	T20N	T20N
	P26S	P26S	P26S	P26S	P26S	P26S	P26S	P26S	P26S	P26S	P26S
	D138Y	D138Y	D138Y	D138Y	D138Y	D138Y	D138Y	D138Y	D138Y	D138Y	D138Y
	R190S	R190S	R190S	R190S	R190S	R190S	R190S	R190S	R190S	R190S	R190S
	K417T	K417T	K417T	K417T	K417T	K417T	K417T	K417T	K417T	K417T	K417T
	E484K	E484K	E484K	E484K	E484K	E484K	E484K	E484K	E484K	–	E484K
	N501Y	N501Y	N501Y	N501Y	N501Y	N501Y	N501Y	N501Y	N501Y	–	N501Y
	D614G	D614G	D614G	D614G	D614G	D614G	D614G	D614G	D614G	D614G	D614G
	H655Y	H655Y	H655Y	H655Y	H655Y	H655Y	H655Y	H655Y	H655Y	H655Y	H655Y
	E661D	E661D	E661D	E661D	E661D	E661D	E661D	E661D	–	–	–
	T1027I	T1027I	T1027I	T1027I	T1027I	T1027I	T1027I	T1027I	T1027I	T1027I	T1027I
	V1176F	V1176F	V1176F	V1176F	V1176F	V1176F	V1176F	V1176F	V1176F	V1176F	V1176F

Corresponding identifiers in the GISAID database EP_ISL_2759001 (A08); EP_ISL_2759002 (A09); EP_ISL_2759011 (C09); EP_ISL_2759016 (C10); EP_ISL_2759036 (D01); EP_ISL_2759037 (E02); EP_ISL_2759044 (E03); EP_ISL_2759058 (E09); EP_ISL_1534008 (Gamma-SC); EP_ISL_1064736 (Gamma-AM) and EP_ISL_2086592 (Gamma-RS)

## Discussion

In this work, we performed an analysis of the evolution of the SARS-CoV-2 pandemic in the state of Paraná, southern Brazil. Our study was based on 333 genomes identified between March 2020 and April 2021 by the Genomic Surveillance Network from Fiocruz and other sequences available in the GISAID database (Additional file 8: Appendix S1). As in other Brazilian states, the year 2020 was marked by the predominance of the variants B.1.1.28 and B.1.1.33 and, from August 2020 onwards, by the VOI P.2 [3, 4]. From January 2021 and in subsequent months, the VOC Gamma emerged in the state and spread fast, reaching 87.2% of all genomes in April 2021. No other variant introduced in Brazil has thus far reached such high prevalence values in the state of Paraná. This may be related to the low social isolation rates observed in Paraná, as well in Brazil, during the COVID-19 pandemic [29–31]. Our analysis reveals that the Gamma variant, first identified in January 2021 in Japanese travelers returning from a trip to the Amazon [5], was already circulating in the state of Paraná by that time.

Additionally, in January 2021, genomes of the VOC Alpha, which are dominant in the United Kingdom [32], were identified in the state of Paraná. Despite the presence of two VOCs in the state of Paraná, it is noticeable that variant Alpha did not replace the most frequent variant or even spread in the state and remained at low frequencies. The dominance of the Gamma variant, even in the presence of another VOC, may be related to the fact that Gamma variant infections are associated with higher viral loads in the upper respiratory tract when compared to non Gamma variant patients [8].

A study conducted in the United Kingdom that evaluated the relationship between the Alpha variant and case severity estimated that the risk of death associated with Alpha is 61% higher than that associated with pre-existing non-VOC lineages [33]. In this study, the presence of the Alpha variant was confirmed by PCR based on S gene amplification failure instead of the complete genome. In our study, we analyzed whether the incidence of the Gamma variant would be related to different age groups or clinical condition strata based on a specific cohort composed of 86 samples from April 2021 that were completely sequenced. Our results did not reveal an association between the Gamma variant prevalence, age range and severity. The Gamma variant was dispersed in all groups with similar severity indices; however, we emphasize that in our April cohort, the Gamma-like-II lineage was identified only in patients under the age of 60 years.

We evaluated the severity of the gamma variant by analyzing samples from a cohort of 86 patients. These samples were classified based on the laboratory of origin. From the LACEN lab oratory, samples were obtained from patients who needed to be admitted to a hospital. From the LAC, outpatient samples were obtained and represent mild cases. According to this specific study, the gamma variant is not associated with severity (*p* value 0.75). Therefore, we infer, based on the available data, that the Gamma variant may not be related to a higher severity of COVID-19, although further studies should be carried out to carefully explore this issue. A limitation of our study includes the possibility of follow-up the clinical outcome of the patients in the cohort.

Our phylogenetic analysis performed with the Gamma variant genomes identified in the state of Paraná and in the other states of Brazil showed that the recently described lineage Gamma-like-II [11] was also present at moderate frequencies in the state of Paraná (Figs. 3 and 5). The Gamma-like-II lineage presents 15 of the 22 mutations of the Gamma variant, including the three main mutations in the RBD domain of the S protein: K417T, E484K and N501Y. On the other hand, they present some unique substitutions: ORF1ab:C8905T, C16954T and A20931G; NSP4:D2980H, intergenic region E/M A26492T and N:P383L. The analysis of the mutations in the first 11 Gamma-like-II genomes from Paraná identified 3 of the 4 unique substitutions of the Gamma-like-II lineage (C8905T, C16954T, A26492T) in addition to the N:P383L and ORF1a:D2980H mutations. However, we also identified some unique substitutions, which were not yet described, for the Gamma variant and Gamma-like-II lineage, and these substitutions in cluded ORF1a:P1213 L and ORF1b:K23240N, which appeared in 45% of the genomes (Additional file 7: Table S2). Our results, in agreement with the initial study [8, 11], show that the VOC Gamma and the Gamma-like-II lineages diverged from a common ancestor that contained some, but not all, of the defining mutations found in the Gamma variant. Among the main mutations common between the two groups, we highlight those that occur in the S protein: S:E484K, S:N501Y and S:D614G. This hypothesis also justifies the absence of other mutations in the S protein, such as S:T20N, in the Gamma-like-II lineage. Our phylogenetic analysis over time showed that the Gamma-like-II lineage emerged between 2 and 4 months later than the Gamma variant. Interestingly, unlike the VOC Gamma, the Gamma-like-II lineage genomes were not dispersed throughout the state of Paraná but were clustered in western Paraná (represented by the host cities, Toledo and Cascavel). Particularly in this region, we found the lowest VOC Gamma frequency of the state, while the Gamma-like-II lineage genomes accounted for almost 30% of the samples sequenced in the cohort study in April 2021. Conversely, the phylogenetic analysis supports a local transmission of the Gamma-like-II lineage in Paraná. To date, Santa Catarina is the Brazilian state where the largest number of the Gamma-like-II lineage genomes has been identified: 30 in total, which corresponds to 71% of the genomes of that state that were previously classified as the Gamma variant by WHO/PANGO (Fig. 5). Of this, 20 Gamma-like-II representatives were identified in the city of Chapecó, which is also located in the western region, approximately 360 km from Toledo and Cascavel. For instance, in February 2021, Chapecó was responsible for the largest outbreak of COVID-19 in the state of Santa Catarina [34]. According to our tree-scaled analysis (Fig. 4), the emergence of the Gamma-like-II lineages in the state of Paraná is estimated to occur at the same time as the appearance of the Gamma-like-II lineages in Santa Catarina. On the other hand, the Gamma-like-II lineages from the other states (São Paulo, Minas Gerais, Alagoas, Espírito Santo) appeared after the variants observed in Paraná and Santa Catarina. These data, together with genomic surveillance information, reinforce the hypothesis that the Gamma-like-II lineage may have arisen in the western area of the state of Santa Catarina. It is possible that the Gamma-like-II lineage may have emerged during the outbreak in the city of Chapecó and initially spread to the western region of Paraná, which could explain the localized expansion of the Gamma-like-II lineage. This result suggests that, unlike the Alpha variant that failed to establish a prevalence in the state and despite having emerged after the VOC Gamma, the constellation of mutations of the Gamma-like-II lineage allowed it to increase in frequency and then cocirculate with the Gamma variant in some specific geographic regions. During the phylogenetic analysis, we noticed that a group formed by 8 genomes from the state of Paraná stood out. Analysis of this group of genomes, classified as belonging to the Gamma variant, revealed the presence of an additional mutation in the S protein (S:E661D). A genomic surveillance study carried out in the United Kingdom identified the S:E661D mutation in viral genomes; however, they were at an extremely low frequency, because 6 genomes out of 142,859 were identified [35]. In our study, the E661D mutation appeared at a much higher frequency and occurred in the Gamma variant, which already has a high number of mutations. We also looked for the presence of this mutation in other Gamma variant genomes from Brazil deposited in the GISAID. We verified the existence of 46 Brazilian Gamma variant genomes with the S:E661D mutation, 20 of which were identified in April 2021. However, our attention was drawn to the high frequency of this mutation in genomes in the state of Paraná, which occurred in 11.35% of the Gamma variant genomes. In comparison, the second state with the highest frequency of the S:E661D mutation was Minas Gerais, with 2.94%. Notably, the S:E661D mutation was not detected throughout the state of Paraná but was observed specifically in northern Paraná, as well as in the capital Curitiba, which accounted for nearly 25% of the Gamma variant genomes. Conversely, the phylogenetic analysis revealed clusterization of the Gamma genomes harboring S:E661D mutation as a function of the state from which the samples were collected, indicating that this mutation had not occurred randomly, but this mutation happened systematically in some geographical regions (Fig. 5).

Cheng and coworkers [36] used structure-based computational models to demonstrate that the SARS-CoV-2 S protein exhibits a high affinity motif for binding to T cell receptors [36]. This predicted motif extends from the amino acid residues E661 to R685 of the S protein. Despite the amino acid change, the E661D mutation maintained the acidic character of the motif, suggesting that this feature may be important for the function of the protein. Additionally, the comparison of the predicted superantigenic region of the SARS-CoV-2 S protein with the same region within bacterial superantigenic proteins showed that there is conservation of aspartic acid residues in bacterial proteins. Although there is no evidence regarding this additional mutation on any aspect of the biology of the virus, it is noteworthy that mutations in the S protein are frequently associated with altered viral fitness and/or immune evasion. For example, the D614G substitution enhanced viral stability and infectivity [37], and the E484 mutation in the receptor-binding domain (RBD) conferred a partial resistance of SARS-CoV-2 to neutralizing antibodies [38]. Our results reinforce the importance of genomic vigilance to track possible novel VOIs that may arise from the current scenario of low vaccination rates and uncontrolled dissemination of SARS-CoV-2 in Brazil, as previously documented [39]. In addition, there are currently 11 Gamma sublines that are accumulating new mutations near the furin cleavage site, among them the E661D described in this work [40].

The classification of genomes into variants performed by Nexclade and Pangolin revealed the presence of an N.10 variant genome in the state of Paraná (N.10-PR). Variant N.10, identified in samples from the Brazilian state of Maranhão, is considered a VOI due to the set of mutations it carries, mainly in the S protein (26). However, unlike the N.10 variant originally described (N.10-MA), the N.10-PR variant has the S:W152C mutation in addition to the other mutations previously described. The S:W152C mutation was initially described in the VOI B.1.429 (CAL.20C), which was identified in the state of California, United States (Additional file 6: Table S1). The importance of the S:W152C mutation is the fact that the NTD is a target for neutralizing antibodies [41]. The VOI B.1.429, which also has a very important S:L452R mutation [38], was identified in California, and to date, there are no reports that it has arrived in Brazil [42, 43]. The other mutations identified in the B.1.429 variant, with the exception of S:D614G, do not match the N.10-PR variant. These results suggest that the N.10-PR S:W152C mutation occurred independently in this variant and has no relation with the VOI B.1.429. The B.1.429 variant has been extensively studied for its infective capacity and binding to neutralizing antibodies. The infective capacities of mutations occurring in the B.1.429 variant were investigated using pseudoviruses carrying the D614G mutation together with the L452R or W152C mutations infected in 293 T cells expressing the ACE2 receptor as well as the cofactor TMPRSS2 [44]. Although the results show an increase in infectivity close to 20-fold for the D614G/L452R protein, the increase in infectivity for D614G/W152C was fourfold when compared to the S protein containing only the D614G mutation [44]. A neutralizing antibody activity study using convalescent serum from patients or serum from vaccinated individuals showed a 3–sixfold reduction against the B.1.429 variant, and furthermore, the neutralizing activity of mAbs targeting NTD was completely blocked due to the combined mutations S:S13I and S:W152C in that domain [43]. Additional information from the GISAID (GISAID—2021–06-01) showed that the S:W152C mutation emerged 41,272 times (2.38% of all samples with spike sequences) in 39 countries. The first strain with this change, collected in July 2020, was hCoV-19/Mexico/ROO-InDRE 243/2020. The most recent occurred in strain hCoV-19/USA/KS KHEL-1975/2021, collected in May 2021, but none of them were identified in Brazil according to the GISAID data. The concern with this N.10-PR variant is the emergence of the S:W152C mutation, and several studies have already showed its relevance to the biology of SARS-CoV-2 due to its S protein that has already accumulated important mutations such as S:V445A, S:E484k and S:D614G. At this point, the N.10-PR variant has as many mutations in the S protein as the VOCs Alpha and B.1.645, which are seven in total.

## Conclusion

A genomic survey from March 2020 onwards in the state of Paraná state, which is based on 333 SARS CoV-2 genomes, allowed us to map the entire dynamics of detection frequency of variants in the state of Paraná since the beginning of the pandemic. This work represents the most complete SARS-CoV-2 genomic surveillance study carried out in the state of Paraná. A phylogenetic analysis revealed that a group of genomes branched into a monophyletic clade, identified as the Gamma-like-II lineage, which probably emerged between February and March 2021. The Gamma-like-II lineage was proportionally more frequently detected in the state of Santa Catarina, which borders the state of Paraná. Interestingly, unlike other lineages, the prevalence of the Gamma-like-II variant in Paraná was not negatively affected by the advancement of the Gamma variant during the period analyzed. We also detected the occurrence of an additional mutation S:(E661D) in the Gamma variant genomes. Finally, having the laboratory of origin of the samples as a parameter, we did not detect a relationship between the Gamma variant, age group and case severity.

## Supplementary Information

**Additional file 1: Figure S1**. SARS-CoV-2 variants identified in Paraná state in April 2021.

**Additional file 2: Figure S2**. Distribution of SARS-CoV-2 variants identified in April 2021, according to macroregions of Paraná state.

**Additional file 3: Figure S3**. SARS-CoV-2 variants identified in Paraná state in April 2021 by age group.

**Additional file 4: Figure S4**. SARS-CoV-2 variants identified at Paraná state in April 2021 by clinical severity at the moment of sample collection (LAC: mild cases; LACEN: severe cases).

**Additional file 5: Figure S5**. The picture shows a time-stamped phylogeny of the B.1.1.28 lineage, Gamma and Gamma like-II lineages in the State of Paraná. In the figure, the length of the branches corresponds to years and the number of mutations, and these are colored according to the legend of colors. In addition, the Gamma genome from the state of Amazonas, the Gamma genomes from Paraná and the group of Gamma-like-II sequences are highlighted. List S1: Number and source of SARS-CoV-2 genomes used in this study.

**Additional file 6: Table S1**. Nonsynonymous mutations described for VOIs N.10-PR, N.10-MA and B.1.429.

**Additional file 7: Table S2**. Nonsynonymous mutations described for Gamma-like-II.

**Additional file 8: Appendix S1**. GISAID acknowledgment file.

## Data Availability

The data are publicly available at NCBI with GenBank accession numbers: MZ477744–MZ477859. The data are available at GISAID with accession numbers: EPI_ISL_2775450-EPI_ISL_2775468 and EPI_ISL_2759020-EPI_ISL_2758965.

## Abbreviations

SARS-CoV-2: Severe Acute Respiratory Syndrome Coronavirus 2
WGS: Whole Genome Sequencing
COVID-19: Coronavirus Disease 2019
VOC: Variant Of Concern
LACEN-PR: The Central Laboratory of the State of Paraná
LAC: COVID-19 Diagnostic Support
OR: Odds Ratio
CI: Confidence Interval
PR: State of Paraná
Kb: Kilobase
SNPs: Single Nucleotide Polymorphisms
MCMC: Markov Chain Monte Carlo
MCC: Maximum Clade Credibility
